# Communication processes about predictive genetic testing within high-risk breast cancer families: a two-phase study design

**DOI:** 10.1038/s41598-021-98737-8

**Published:** 2021-10-11

**Authors:** Chiara L. Blomen, Aliaksandra Pott, Alexander E. Volk, Lars Budäus, Isabell Witzel

**Affiliations:** 1 Department of Gynecology, University Medical Center Hamburg-Eppendorf, Martinistraße 52, 20246 Hamburg, Germany; 2 Department of Medical Psychology, University Medical Center Hamburg-Eppendorf, Hamburg, Germany; 3 Institute of Human Genetics, University Medical Center Hamburg-Eppendorf, Hamburg, Germany; 4 Martini-Klinik Prostate Cancer Center, University Medical Center Hamburg-Eppendorf, Hamburg, Germany

**Keywords:** Patient education, Quality of life, Cancer genetics, Clinical genetics, Medical genetics, Breast cancer, Cancer genetics, Cancer, Genetics, Psychology, Health care, Medical research, Oncology

## Abstract

The detection of a pathogenic variant in the *BRCA1* or *BRCA2* gene has medical and psychological consequences for both, affected mutation carriers and their relatives. A two-phase study with explanatory sequential mixed methods design examined the psychological impact of genetic testing and associated family communication processes. Analyzing a survey data of 79 carriers of a *BRCA1* or *BRCA2* mutation, the majority had general psychological distress independent of cancer diagnosis in the patients’ history. The point prevalence of depression was 16.9%. Contrary to their subjective perception, the respondents’ knowledge about those mutations was moderate. Despite the high rate of information transfer to relatives at risk (100%), their reported uptake of genetic testing was low (45.6%). Communication about the mutation detection was more frequent with female than with male relatives. In-depth focus group interviews revealed significant barriers to accessing genetic counseling including anxiety, uncertainty about the benefits of testing and about the own cancer risk, particularly among males. This study suggests that an adequate knowledge of the genetic background and psychological support is required to reduce emotional distress, to support familial communication and to facilitate genetic testing.

## Introduction

Approximately 30% of all breast cancer patients have a family history of cancer^1^. About 5–10% of all breast cancers are hereditary, mostly resulting from pathogenic variants in the tumor suppressor genes *BRCA1* or *BRCA2*^2^. Genetic testing is indicated if a family history of cancer implies an elevated risk of a *BRCA1* or *BRCA2* mutation and aims to prevent carcinogenesis through intensified screening programs or prophylactic surgeries^3^.

In addition to medical consequences, the detection of a mutation in a cancer predisposing gene also has psychosocial impact. It can be perceived as a psychological burden by some mutation carriers^4–6^, not only resulting from uncertainties and fears concerning the own future prospects, but also from the responsibility to inform relatives about their individual risk to carry the same mutation and its associated cancer risks. In order to advise family members, a mutation carrier has to understand the test result and its clinical consequences. Thus, knowledge about the gene alteration significantly influences the process of disclosing a pathogenic test result to relatives^7^. In particular, the complex nature of genetics and associated uncertainties about the families’ risk may act as a barrier in the communication process^7,8^, whereas valid knowledge about the gene alteration may enhance family communication^9^. Moreover, family-related factors have impact on communication^10^, for instance the presence of a family history of cancer, the quality of intrafamilial relationships^11,12^ as well as the communication style within a family. Furthermore, communication may be affected by emotional barriers, for instance the mutation carriers’ desire to protect relatives from the psychosocial distress associated with that hereditary cancer risk^8^. In addition, there might be a discrepancy in the communication frequencies with male and female relatives, thus partially underestimating the relevance for males^13^. Culture-based factors can also influence the communication process^10^. In conclusion, the disclosure of familial cancer risk to relatives is influenced multifactorially^7,10^. However, the subsequent uptake of predictive *BRCA1* or *BRCA2* testing by relatives at risk is also dependent on sociodemographic, economic, and psychosocial variables on the family members’ side^14^. In this study we investigated how the disclosure of genetic test results by *BRCA1* or *BRCA2* mutation carriers influences the uptake of consecutive genetic testing of relatives at risk.

## Methods

A two-phase study with explanatory sequential mixed methods design^15^ was conducted including a quantitative study phase (ten-page questionnaire) with *BRCA1* or *BRCA2* mutation carriers and a qualitative study phase (two focus group interviews) with relatives and partners. Ethical approval was obtained from the board of the medical ethics committee of Hamburg (number: PV6060). All methods were carried out in accordance with relevant guidelines and regulations. Informed consent was obtained from all participants.

### Quantitative study part: questionnaire survey

#### Participants and methods

**Figure 1:**
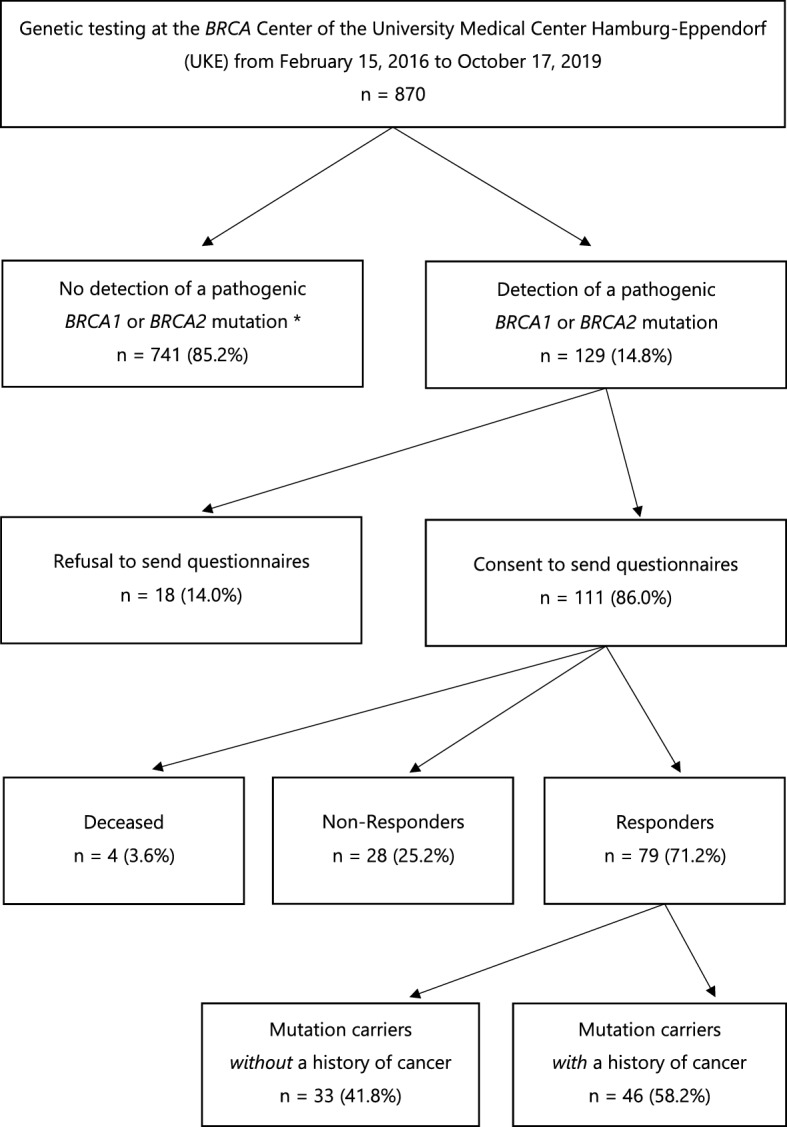
Consort diagram. *Includes tests with inconspicuous results, detection of other mutations, variants of unclear significance.

Between February 2016 and October 2019 870 patients had undergone genetic counseling and testing at the University Center of Hereditary Breast and Ovarian Cancer according to the guideline of the German Consortium for Hereditary Breast and Ovarian Cancer (GC-HBOC)^3^. The median time between genetic testing and questionnaire was 13.0 months (range 2–42). A pathogenic variant in the genes *BRCA1* or *BRCA2* was found in 129 cases (15%), which were recruited for the study. Inclusion criteria were knowledge of the German language, age of 18 years, and agreement to receive questionnaires for scientific purposes (n = 111). A total of 79 respondents answered the questionnaire completely, resulting in a response rate of 71.2% (Fig. 1).

#### Variables and instruments

The questionnaire included sociodemographic items, mutation carriers’ history of cancer as well as the family history of cancer. Furthermore, the psychological well-being of mutation carriers was explored by validated instruments: general distress via the *NCCN Distress Thermometer*^16^, depression via the *Patient Health Questionnaire-9 (PHQ-9*)^17^, anxiety via the *Generalized Anxiety Disorder-7 (GAD-7*)^18^ and health-related quality of life via the *Short Form-12 (SF-12*)^19^. The *NCCN Distress Thermometer* is a short screening instrument recording general psychosocial distress of many possible causes in oncological patients, consisting of a scale from 0 to 10, graphically represented as a thermometer. Internationally, a cut-off value of 5 is recommended as a signal that a person is noticeably stressed or needs support^16^. Furthermore, questions regarding genetic testing were asked: reasons for genetic testing via an *eight validated item scale*^20^, satisfaction with the decision to undergo genetic testing via the *Satisfaction with Decision Scale (SWD Scale*)^21^, the psychological impact of genetic testing via the *Impact of Event Scale (IES)*^22^ and the knowledge about the genetic alteration via the *Breast Cancer Genetic Counseling Knowledge Score (BGKQ)*^23^. Based on scientific research, nine questions were developed for communication with family members and six questions regarding the information and support needs of mutation carriers.

#### Statistical analyses

Descriptive statistics were calculated to present demographic characteristics. Relative frequencies and means (M) including standard deviations (SD) were calculated. For subgroup analysis of two independent groups, nonparametric inferential statistical test procedures (Mann–Whitney U test) were performed. A p-value of less than 0.05 was regarded as statistically significant. All analyses were performed using Statistical Package for the Social Sciences (IBM SPSS Statistics version 25, IBM Corporation, Germany).

### Qualitative study part: focus group interviews

The objective of the qualitative survey was to gain a more in-depth understanding of communication processes in families with hereditary cancer predisposition and to identify needs on the part of family members of mutation carriers.

When questionnaires were sent to mutation carriers by letter, also information material about the qualitative study part were provided. Mutation carriers were asked to pass on these materials to two family members of their choice who could then sign up for the interviews. Because of 10 written informed consents for the focus group, only two semi-structured interviews were conducted in August 2020 with six relatives (four female, two male) and four partners (one female, three male).

The interview guide for the focus groups based on previously defined research questions (Supplementary Fig. S1). The interviews lasted 60 minutes and were moderated by two research members (A.P. and C.B.). The participants filled out an one-page questionnaire about personal data. Finally, the prepared questions were asked to examine personal experiences of the family members and to understand the different perspectives on genetic testing. The participants were asked to describe their experiences in detail. The complete interviews were audio-recorded. Data were anonymised and thematically transcribed by two research members (A.P. and C.B.). The final transcripts of 30 pages were analyzed using MAXQDA20 software (VERBI GmbH, Berlin, Germany) and following the concept of content analysis^24^. Two team members (A.P. and C.B.) discussed and independently coded the transcripts. Most of the categories showed high consistence. The categories were discussed in the research team avoiding missing the clarity of categories. For the intent analysis all information of the transcripts were used.

### Ethical approval

Ethical approval was obtained from the board of the medical ethics committee of Hamburg (PV6060, 18.6.2019).

## Results

### Quantitative study part

#### Sample characteristics

The mean age of respondents was 48 years (range 21–77) and the sample consisted of mostly female participants (84.8%), living in a relationship (75.9%), with at least one other person in the household (86.1%), and at least one child (72.2%). Most respondents reported having at least one sibling (83.5%). Regarding educational status, the majority had a higher education level (62.8%). More than half of the mutation carriers had a history of cancer (58.2%). Of these, breast cancer was the most common, followed by ovarian cancer. Table 1 presents further sociodemographic characteristics of the study sample.

**Table 1:** Sociodemographic and clinical characteristics of the sample.

Sociodemographic and clinical characteristics	n (%)	Cumulated %
**Gender**		
Female	67 (84.8)	84.8
Male	12 (15.2)	100
**Marital status**		
Married	48 (60.8)	60.8
Committed partnership, unmarried	12 (15.2)	75.9
Single	10 (12.7)	88.6
Divorced or separated	7 (8.9)	97.5
Widowed	2 (2.5)	100.0
**Living situation**		
With other persons	68 (86.1)	86.1
Alone	11 (13.9)	100.0
**Children**		
No children	22 (27.8)	27.8
1 Child	23 (29.1)	57.0
2 Children	26 (32.9)	89.9
> 2 Children	8 (10.2)	100.0
Of these minors (< 18 years)	36 (63.2)	
**Siblings**		
No siblings	13 (16.5)	16.5
1 Sibling	35 (44.3)	60.8
2 Siblings	22 (27.8)	88.6
> 2 Siblings	9 (11.4)	100.0
At least 1 sister	45 (57.0)	
At least 1 brother	36 (45.6)	
**Graduation**		
Lower school-leaving qualification	29 (37.2)	37.2
Higher educational level	49 (62.8)	100.0
**Professional career**		
Apprenticeship, Bachelors Degree	44 (55.7)	55.7
University, technical college	29 (36.7)	92.4
Other	6 (7.6)	100.0
**Work situation**		
Employed	49 (62.0)	62.0
Not employed	18 (22.8)	84.8
Other	12 (15.2)	100.0
**History of cancer**		
Yes	46 (58.2)	58.2
No	33 (41.8)	100.0
**Tumor entities**		
Breast cancer only	27 (58.7)	58.7
Ovarian cancer only	11 (23.9)	82.6
Breast and ovarian cancer	4 (8.7)	91.3
Breast and additional cancer	2 (4.4)	95.7
Other cancer entity	2 (4.4)	100.0

In 86.1% of participants, there was a family history of cancer, with an average of 2.79 (SD = 1.64) tumor cases per family. The most frequently affected relatives were mothers (55.7%), aunts (38.0%), grandmothers (35.4%) and fathers (24.1%). There was a prevalence of breast cancer in 70.9% of families, of ovarian cancer in 29.1%, of prostate cancer in 16.5% and of colorectal cancer in 12.7% of families (Table 2).

**Table 2:** Descriptive statistics of family history of cancer.

Characteristics	n (%)
**Family history of cancer**	
Yes	68 (86.1)
No	11 (13.9)
**Family members with cancer**	
Mother	44 (55.7)
Father	19 (24.1)
Sister	10 (12.7)
Brother	6 (7.6)
Aunt	30 (38.0)
Uncle	12 (15.2)
Cousin (female)	13 (16.5)
Cousin (male)	0 (0.0)
Grandmother	28 (35.4)
Grandfather	7 (8.9)
Tumor entities*	
Breast cancer	56 (70.9)
Ovarian cancer	23 (29.1)
Colorectal cancer	10 (12.7)
Prostate cancer	13 (16.5)

*Refers to the general presence of a specific tumor entity within a family. It is possible that several family members were affected by this type of cancer.

#### Psychological well-being of mutation carriers

Table 3 shows psychological variables of *BRCA1* or *BRCA2* mutation carriers concerning distress, depression, anxiety and health-related quality of life. Measured by *NCCN Distress Thermometer* (cut-off ≥ 5), 64.6% of participants had general distress (n = 51, M = 5.37, SD = 2.3, Table 3). No difference in general distress was found between participants without and with a history of cancer (69.7% vs. 60.9%, *p* = 0.423, Table 3). The point prevalence of depression (*PHQ-9*, cut off ≥ 10) was 16.9% (n = 13, Table 3). Only a minority of participants had moderate or severe depressive symptoms (14.3% or 2.6%, respectively) (Table 3). There was no significant association between history of cancer (*p* = 0.473), gender (*p* = 0.287), living situation (*p* = 0.156), having children (*p* = 0.513) or educational degree (*p* = 0.059), and severity of depression (data not shown). Measured by *GAD-7*, mild or moderate anxiety symptoms were present in 47.4% (n = 37) and severe anxiety symptoms in 2.6% (n = 2) of participants (Table 3) with no difference regarding history of cancer (*p* = 0.696, data not shown). There was a deviation of about one standard deviation in the health-related quality of life (*SF-12)* in the physical and mental domain compared with the norm mean values of 41- to 50-year-olds in the general population which means slight restrictions in the daily functional level of participants^25^ (Table 3).

**Table 3:** Variables of psychological well-being.

Variables		n (%)	M (SD)
NCCN distress thermometer*			
No		28 (35.4)	
Yes		51 (64.6)	
Distress, population without cancer		23 (69.7)	
Distress, population with a history of cancer		28 (60.9)	
Patient health questionnaire 9 (PHQ-9)**			
No		64 (83.1)	
Yes		13 (16.9)	
0–4	Minimal	33 (42.9)	
5–9	Mild	31 (40.3)	
10–14	Moderate	11 (14.3)	
15–19	Moderately severe	0 (0)	
20–27	Severe	2 (2.6)	
**Generalized anxiety disorder-7 (GAD-7)**			
0–4	Minimal	39 (50.0)	
5–9	Mild	30 (38.4)	
10–14	Moderate	7 (9.0)	
15–21	Severe	2 (2.6)	
Short form-12 (SF-12)***			
Physical domain			46.96 (10.11)
Mental domain			47.66 (10.09)

*Cut-Off ≥ 5 general distress.

**Cut-Off ≥ 10 major depression.

***In comparison, the norm means of 41- to 50-year-olds in the general population (1994) were M = 50.15 (SD = 7.93) for the physical domain and M = 52.24 (SD = 7.79) for the mental domain.

#### Genetic testing

Measured by an *eight validated item scale* main reasons for undergoing genetic testing were desire for safety, prevention and risk assessment for the own children. Satisfaction with the decision *(SWD)* to undergo genetic testing was high (Table 4).

The subjective distress related to genetic testing *(IES)* showed an inconspicuous result in 59.5% (n = 47), a moderate impact in 30.4% (n = 24) and a severe impact in 10.1% (n = 8) of participants (Table 4). Psychosocial support because of genetic testing and related concerns was used by 41.8% (n = 33) of respondents, in particular psychosocial counseling services (20.5%, n = 16), psychotherapy (16.5%, n = 13) as well as internet forums with other affected individuals (16.5%, n = 13). Especially psychotherapy and psychosocial counseling services were perceived as helpful. Overall, *BRCA1* or *BRCA2* mutation carriers felt well to very well informed about the purpose of genetic testing, the test result and the impact on their family (Table 4). Further information needs were expressed through individual comments and related mainly to the following topics: ovarian cancer, prophylactic and preventive measures, life after prophylactic surgery and concrete instructions for follow-up. The objectively measured knowledge (*BGKQ*) about the gene alteration was moderate. On average, M = 16.47 (SD = 5.08) of 27 items (Median = 18, range 1–26) were answered correctly (Table 4). The results of items regarding genetic testing are presented in Table 4.

**Table 4:** Analyzed factors of genetic testing.

Genetic testing		M (SD)	n (%)
Reasons to attend genetic counseling*			
To obtain certainty		4.49 (0.94)	
To be able to take preventive actions		4.40 (1.02)	
To estimate the risk for my children		3.77 (1.56)	
To help science		3.18 (1.40)	
Requested by a family member		2.19 (1.42)	
General planning for the future		2.73 (1.48)	
Family planning		1.68 (1.27)	
Other		1.52 (1.39)	
Satisfaction with decision to undergo genetic testing*			
Sum score**		27.27 (4.19)	
I am satisfied that I am informed about the issues important to my decision		4.60 (0.74)	
The decison I made was the best decision possible for me personally		4.56 (0.68)	
I am satisfied that my decision was consistent with my personal values		4.61 (0.74)	
I expect to successfully carry out the decision I made		4.53 (0.88)	
I am satisfied that this was my decision to make		4.52 (0.95)	
I am satisfied with my decision		4.56 (0.86)	
Impact of Event Scale (IES)***			
≥ 25	Inconspicuous		47 (59.5)
26–43	Moderate impact		24 (30.4)
> 43	Severe impact		8 (10.1)
Subjective perception of own knowledge*			
Informed about the significance of genetic testing		4.51 (0.72)	
Informed about advantages and disadvantages		4.25 (0.78)	
Informed about the test procedure		4.24 (0.96)	
Informed about the risks of genetic testing		3.86 (1.21)	
Informed about the test results		4.47 (0.62)	
Informed about the consequences for relatives		4.22 (0.97)	

			Median (min–max)
**Objective knowledge (breast cancer genetic knowledge counseling score, BGKQ)**			
Correct answers****		16.47 (5.08)	18 (1–26)

*These items could be answered from 1 “absolutely not agree” to 5 “totally agree”.

**Sum Score of *SWD Scale*: Maximum of 30 is equivalent to full satisfaction with undergoing genetic testing.

***Impact of Event Scale measures the subjective distress related to a specific event such as genetic testing.

****A total of 27 items had to be answered.

#### Communication about mutation detection within families

**Figure 2:**
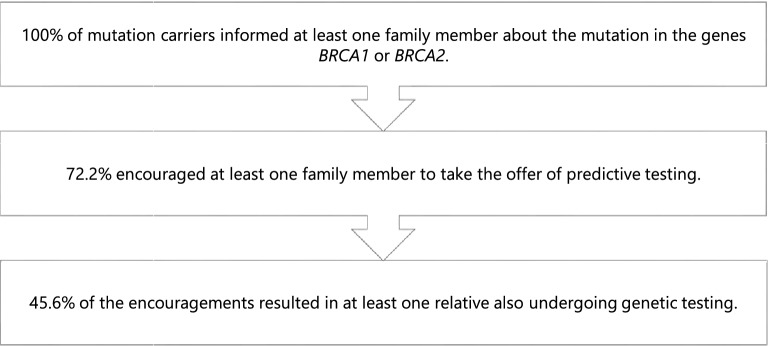
The familial communication process and following uptake of genetic testing.

All respondents informed at least one family member about the detection of a mutation and 72.2% (n = 57) encouraged at least one relative to undergo predictive genetic testing. A recommendation to genetic testing was finally followed in 45.6% (n = 26) of the families. The familial communication process and following genetic uptake are summarized in Fig. 2

.

Most frequently, the sister (88.9%, n = 40) was informed about the gene alteration (Table 5). The brother was informed by 75.0% (n = 27) of participants. Overall, in each comparable group, communication about the mutation detection was more frequent with female than with male relatives. Furthermore, 64.9% (n = 37) of respondents informed their children about the gene alteration (Table 5).

**Table 5:** Communication partners within families.

Communication partners	n (%)	Total n
Child	37 (64.9*)	57
Mother	37 (46.8)	79
Father	28 (35.1)	79
Sister	40 (88.9*)	45
Brother	27 (75.0*)	36
Aunt	20 (32.2*)	59
Uncle	9 (17.6*)	51
Cousin (female)	23 (29.1)	79
Cousin (male)	3 (3.8)	79

*Percentage in relation to total frequency.

The majority (57.0%, n = 45) informed their relatives immediately after receiving the test result or one to two days later (36.7%, n = 29). Further discussions also took place after seven to ten days (24.1%, n = 19) or after weeks to months (25.3%, n = 20).

### Qualitative study part

The in-depth focus group interviews with six relatives and four partners were evaluated. Participating family members had become aware of genetic testing either through a history of cancer of a (female) family member or through their own cancer disease. In general, all families communicated about the detection of a pathogenic variant and in most cases the possibility of predictive testing was mentioned. Finally, family members responded differently to familial predisposition to cancer: *Fact-oriented* reactions concerning the familial *BRCA1* or *BRCA2* mutation were observed in individuals with an own history of cancer. They described a process of adaptation which was the result of continuous confrontation with oncological diseases within a family. Contact to health care professionals, intensified follow-up, screening programs and prophylactic surgery for mutation carriers had made it possible to develop feelings of security, control and self-efficacy. *Anxious-avoidant* reactions were described by relatives who experienced particularly severe cancer cases in their family or who were unprepared to learn about the familial genetic disposition, both resulting in an information processing under fear. Strong feelings, e.g. feeling helpless, distressed or overwhelmed had been intensified by the lack of detailed information, leading to the avoidance of genetic counseling. *Emotionally-distant* reactions were observed in relatives who were informed about the genetic predisposition during cancer diagnosis and treatment of a family member. In this acute stress situation, family members considered their own predictive testing a lower priority. Together with an uncertainty who to contact for further information, they paid less attention to the own risk and the possibility of having predictive testing.

From the relatives’ perspective the main arguments for undergoing predictive genetic testing were gaining certainty and access to further information and screening programs in case of a pathogenic test result. The main reasons against undergoing predictive genetic testing were young age (< 25 years), fear of the burden associated with a pathogenic test result, family conflicts as well as uncertainty concerning the clinical relevance of testing and the own risk, especially among male relatives. Relatives who had already been tested emphasized that the genetic testing itself had been less challenging, whereas the previous process of decision-making had been emotionally stressful, especially because of uncertainties due to a deficiency of valid information. It was highlighted that family members only got their information about predictive testing from a cancer patient.

## Discussion

In this study, we have shown that *BRCA1* or *BRCA2* mutation carriers have high levels of psychological distress irrespective of a history of cancer. Although participants of this study felt well-informed about genetic testing, their objectively tested knowledge was moderate. Test results were frequently forwarded to relatives at risk. However, only a minority of informed relatives took the offer of predictive testing. Male family members were less integrated into communication processes.

In line with previous research^5,26,27^, our results from the *NCCN Distress Thermometer*, *PHQ-9* and *GAD-7* demonstrated significant psychological distress among *BRCA1* and *BRCA2* mutation carriers, independent of a history of cancer. In a cross-sectional study, the point prevalence for depression in breast cancer patients was 9.3% (95% CI 8.7–10.0%)^28^, whereas the point prevalence for major depression in our sample of mutation carriers was 16.9% (n = 13). Mella et al. observed even higher anxiety and depression levels in *BRCA1* or *BRCA2* mutation carriers without cancer than in diseased individuals^29^. These findings demonstrate that the detection of a mutation can cause cancer fear and lead to psychological distress. However, it should be emphasized that study results dealing with the psychological impact of genetic testing are heterogenous^4,6^, and that other research showed no significant decrease in psychological well-being due to the detection of a pathogenic mutation, especially considering the intermediate- and long-term outcome after test disclosure.

The complex nature of genetic information can make it difficult to understand and remember it correctly, resulting in limited knowledge of mutation carriers^7,30^. Lerman et al. found that only 55% of 11 items concerning the gene alteration were answered correctly^31^. Similarly, in our study, an average of 61% of 27 *BGKQ*-items were answered correctly. The discrepancy between such limited knowledge and feeling (very) well informed about the mutation has impact on disclosing genetic information to relatives and ultimately on their risk perception. Previous research showed that poor understanding and associated uncertainties about the gene alteration, in particular concerning empirical propabilities^32^, made it difficult to communicate with family members^33^. These qualitative deficiencies in information transfer may have contributed, among other factors, e.g. the relevant number of minor children of study participants^12^, to low uptake rates of predictive genetic testing among relatives at risk seen in this study and in previous research^10^.

Taken together, mutation carriers express the need for comprehensive information and supportive consultations with health care professionals^7^. The use of a screening method (for instance, the *NCCN Distress Thermometer*) during genetic counseling would enable to identify vulnerable individuals and to offer them psychosocial support^34^. As mutation carriers were satisfied with psychosocial support, it might also be beneficial to offer it to relatives at risk. Moreover, adjusted information emphasizing the significance of a pathogenic test result also for relatives could facilitate the familial communication process^35^.

The strengths of this study included the comprehensive assessment of psychological well-being of mutation carriers through four different instruments. We further compared mutation carriers with and without a history of cancer. By using a two-phase study design, we were able to analyze the topic of genetic testing efficiently and to gain a deeper insight into the mutation carriers’ and their relatives’ perspective.

A limitation of this study is its’ retrospective design, thus the time of genetic testing and detection of a pathogenic mutation differed among the study participants. This may have led to bias in the analysis. Despite the high rate of psychological distress found in this study, most mutation carriers were very satisfied with the decision of undergoing genetic testing. The presence of psychological distress due to the detection of a mutation is plausible, nonetheless, it should be noted that no causal relationship between psychological distress and genetic testing could be proven in this study. Other factors (for instance, socioeconomic, health-related, family-related) may also have contributed to the respondents’ decline in psychological well-being. However, we could not find a statistically significant impact of the variables *gender*, *educational degree, living situation* or *having children* on depressive symptoms. Also, the results of the *Impact of Event Scale* indicate that genetic testing can cause emotional distress, at least for a significant percentage of *BRCA1* or *BRCA2* mutation carriers. Unfortunately, we had no valid information about the number of prophylactic surgeries which might also have an influence on the individual stress level.

Another limiting factor in our analysis was that men and women were included in the study population, however, middle-aged women were overrepresented, equivalent to previous studies on genetic testing^4,29^. This could also have led to a potential underrepresentation of gender-based aspects. Further studies should focus on the situation of male *BRCA1* or *BRCA2* mutation carriers or on the situation of mutation carriers in other hereditary diseases (e.g. familial colorectal cancer).

Another important limitation is the small number of participants in the focus group interviews, resulting in limited generalizability of our findings.

The different situation of mutation carriers with and without a history of cancer was considered by separate analysis and comparison of the groups. Nevertheless, even among affected individuals, the severity of cancer diagnosis and its treatment differed, which may also have had an impact on psychological distress of the respondents and thus the representativeness of the data. Further studies should focus on additional variables which might have an impact on psychological well-being of mutation carriers and include larger samples of relatives in order to evaluate the needs of those who have not yet decided on undergoing genetic testing. Taken together, our results suggest that genetic testing may have an emotional and psychosocial impact at least on some family members of *BRCA1* or *BRCA2* mutation carriers, and that some of them may require professional support in both cognitive and psychological processing of their relatives’ test results.

## Conclusion

The main finding of our study is that genetic testing is associated with psychological distress and moderate knowledge of genetic test meaning by mutation carriers. Although genetic risk information transfer to relatives at risk was high, their reported uptake of genetic testing was low. To improve the understanding of the value of predictive genetic testing, a closer look at psychological aspects of relatives at risk, especially without a cancer diagnosis, and at males is needed. The involved health care professionals need to be aware of this fact and be able to support the communication process within families by giving profound information about hereditary cancer predisposition.

## Supplementary Information

Supplementary Information.

## Data Availability

The datasets used and/or analysed during the current study are available from the corresponding author on reasonable request.
