# Supplementary material for: Communication processes about predictive genetic testing within high-risk breast cancer families: a two-phase study design
Source: Sci Rep. 2021 Oct 11;11:20178. doi: 10.1038/s41598-021-98737-8 (PMC8505562; doi:10.1038/s41598-021-98737-8)
Supplement: Supplementary file 1 — Supplementary Information. [file 41598_2021_98737_MOESM1_ESM.pdf]

Supplementary Material

Figure S1: Interview guide for the focus groups

Interview guide for the focus groups

- **Assess experience of genetic testing**
    - How did you first learn about the gene mutation and the familial risk?
        - Which family member was affected by the gene mutation?
        - Who informed you and in which context?
    - How did you experience the genetic testing of your family member?
        - Which information did you find useful?
        - How was your own mental state and the family members state?
        - Did you observe supportive or obstructive thoughts or circumstances?

- **Explore communication process**
    - How did your family deal with and communicate about the issue?
        - How was the conversation about familial predisposition?
        - When did the conversations take place?
        - Which issues were addressed specifically?
        - Which information did you get that addressed you as a family member?
        - How did you personally feel during these conversations?
        - Did you feel that the mutation carrier was able to cope with informing you about the gene alteration?
    - What consequences and challenges have you experienced during conversation within the family?
        - Did you get an idea of your own risk?
        - Did you draw any concrete conclusions yourself?
        - Have you undergone genetic counseling and testing after the conversation? If not, which were the reasons why you decided against genetic counseling?

- **Define changing points to optimize communication**
    - What can be done from health care systems' side to facilitate the processing of information on familial genetic alteration and to support positive coping?
    - What can be done to encourage uptake of genetic testing by relatives at risk?
    - What would you expect from optimal health care? (for instance, specific written information material, internet addresses, consultations with psychological professionals)

Note. The interview guide was originally formulated in German and was translated into English for the manuscript.
